# Antimicrobial resistance, virulence factors and phylogenetic profiles of *Vibrio parahaemolyticus* in the eastern coast of Shenzhen

**DOI:** 10.3389/fmicb.2024.1452942

**Published:** 2024-11-11

**Authors:** Xian Qiang Lian, Guo Dong Liu, Miao Fen Huang, Qiu Hua Fan, Zi Dan Lin

**Affiliations:** Dapeng New District Center for Disease Control and Prevention, Shenzhen, Guangdong, China

**Keywords:** *Vibrio parahaemolyticus*, antimicrobial resistance, virulence factors, phylogenetic, SNP (single nucleotide polymorphism)

## Abstract

*Vibrio parahaemolyticus* (*V. parahaemolyticus*) is a major food-borne pathogen which causes human gastroenteritis. Since the characteristics of *V. parahaemolyticus* remain unknown, 220 isolates selected from clinical and environmental samples in Dapeng of Shenzhen were tested for the presence of two hemolysin-expressing genes *tdh* and *trh*. Among 27 clinical isolates, 26 carrired the *tdh* gene, and the other one carried both *tdh* and *trh* genes, however neither genes were detected in environmental isolates. Meanwhile, antimicrobial susceptibility profiles revealed the isolates with high frequency of resistance to ampicillin (77.73%) and colistin (71.82%) and medium to streptomycin (57.27%). Genetically, by whole genome sequencing (WGS), comparative genomics studies was performed on isolates from various districts and GenBank. Data analysis showed that antimicrobial resistance genes (ARGs) *blaCARB*, *tet(34)* and *tet(35)* were harbored in all genomes and other ARGs was absent in the genomes of 27 clinical isolates. Besides, little regional difference was observed. As for virulence factors, MAM7, T3SS1, T3SS1 secret effector, T3SS2, T3SS2 secret effector, and VpadF were carried by most isolates. Two isolates from other districts were *tdh* gene positive which clustered with clinical isolates from Dapeng in the same clade, indicating close genetic distance. This study revealed the widely distribution of *V. parahaemolyticus* in Shenzhen and the diverse ARGs and virulence genes it carried. Furthermore, pathways that pathogen disseminated through were discussed.

## 1 Introduction

*V. parahaemolyticus* is a universal marine microorganism responsible for severe diarrhea in humans (Baker-Austin et al., 2018; Su and Liu, 2007). Predominantly inhabiting in warm seawater along coastlines, its population density significantly increases in summer. Since global warming has been an imminent and non-negligible issue, *V. parahaemolyticus* distribution could be undergoing considerable changes (Baker-Austin et al., 2010). Consequently, human infection and the ensuing gastrointestinal diseases such as diarrhea, nausea, abdominal cramps and even death would affect larger population and threaten the public health of global costal region (Ralph and Currie, 2007). In Asian countries and United States, *V. parahaemolyticus* has been the one of the most common causes of food poisoning and infection cases kept boosting due to climate change and public dietary habits alternation, such as increasing seafood consumption (Letchumanan et al., 2014; Li et al., 2014).

Hemolysin expressed by *tdh* and *trh* genes are the major virulence factors for *V. parahaemolyticus*, which causes hemolysis, cytotoxicity and intestinal toxicity, not to mention other disease-causing genes such as *ure*, *Mtase*, and *Vp-PAI* (Nishibuchi and Kaper, 1995; Nordstrom et al., 2007). Facing excessive use of antibiotics and the ensuing muti-drug resistance (Cabello, 2006; Mazel and Davies, 1999), cephalosporins, tetracyclines, quinolones and fluoroquinolones which were recognized as the first-line antimicrobial treating *vibrio* infections have been challenged by updated bacteria (Jin et al., 2021; Morris and Tenney, 1985). Thus, it elucidates the significance of antimicrobial resistance profile of *V. parahaemolyticus* which lays solid foundation for optimized therapy and drug development (Elmahdi et al., 2016). Besides, WGS offers a high-resolution approach for molecular epidemiological investigations and facilitates the elucidation of evolutionary relationships among isolates aiding in potential origins discovery (Gonzalez-Escalona et al., 2017).

Dapeng district was selected as research site since it harbors extremely long coastline and the city it belongs to, Shenzhen, is a region severely affected by *V. parahaemolyticus* (Li et al., 2014). Less affected by industrialization, isolates from Dapeng were in comparison with those from other regions in Shenzhen, which may give us insight into how environmental pollution affect bacteria genome variation and characteristics such as pathogen resistance (Ansari et al., 2008; Malik and Aleem, 2011), providing instructions for treatment risk prevention and assessment.

In this study, we characterized the profiles of *V. parahaemolyticus* by assessing antimicrobial resistance, virulence genes, and phylogenomic relationships of isolates from clinical and natural sources. Additionally, we compared the clonal lineages, virulence, and resistance genes within *V. parahaemolyticus* genomes from samples of regional difference.

## 2 Materials and methods

### 2.1 Bacterial strains

**Figure 1:**
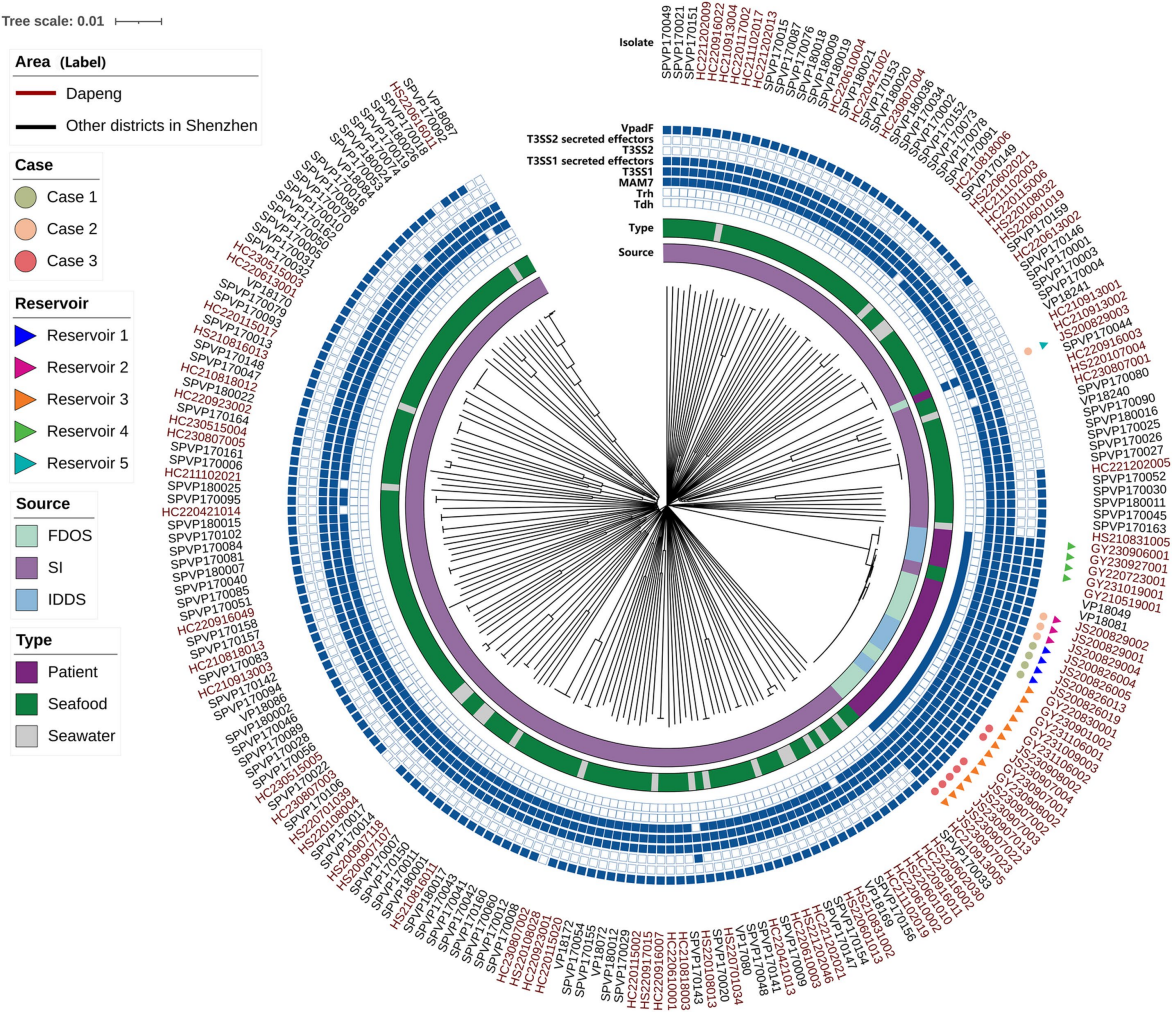
Neighbor-Joining phylogeny based on SNPs of the 218 *Vibrio parahaemolyticus* genomes from patients, seafood, seawater samples of Dapeng district and the environment in other districts of Shenzhen city. The scale bar indicates branch lengths within the tree.

The *V. parahaemolyticus* isolates analyzed in this study (Table 1) had been collected since 2020, over a 4-year period. Among them, 134 were sourced from 160 nearshore seawater samples, and 59 were derived from 146 freshly caught seafood samples in local marine area (Seawater/Seafood-Investigation program, SI). 15 clinical isolates were retrieved from Foodborne-Disease-Outbreak-Surveillance (FDOS), corresponding to three distinct food-borne outbreak cases (Figure 1; Supplementary Table 1). 12 clinical isolates were obtained from Infectious-Diarrhoeal-Diseases-Surveillance (IDDS) program provided by a hospital located in Dapeng. Additionally, the genomic sequences of 123 environmental isolates from other districts were downloaded from NCBI GenBank database (Supplementary Table 1). Previously preserved at −80°C, all isolates were retrieved and then inoculated onto thiosulfate citrate bile-salt sucrose (TCBS) agar plates and incubated at 37°C for 24 h. Isolates were monitored by VITEK^®^ 2 Compact (Merrier) and ATCC17802 was used as the quality control strain. *Tlh* was marked to ensure it could be detected by real-time polymerase chain reaction (PCR).

**Table 1:** Distribution of *Vibrio parahaemolyticus* isolates from different sources used in this study.

Sample type tested	No. of samples	No. of positive samples	Positive percentage
Seafood in total	146	59	40.41%
Fish	47	19	40.43%
Crustaceans	43	14	32.56%
Shellfish	51	22	43.14%
Cephalopods	5	4	80.00%
Patients in total	/	27	/
FDOS	/	15	/
IDDS	/	12	/
Seawater	160	134	83.75%

### 2.2 Determination of antimicrobial resistance

Antimicrobial resistance were determined using the BD Phoenix™ M50. *Escherichia coli* ATCC25922 was used for the quality control. The results were interpreted using the Clinical and Laboratory Standards Institute (CLSI) M100 guideline (CLSI, Clinical and Laboratory Standards Institute, 2024). Antibiotic tested involved chlorphenicol (CHL, 8-32ug/ml), trimethoprim-sulfamethoxazole (SXT, 2/38–4/76ug/ml), colistin (COL, 2-4ug/ml), ertapenem (ETP, 0.5-2ug/ml), meropenem (MEM, 1-4ug/ml), cefotaxime (CTX, 1-2ug/ml), ceftazidime (CAZ, 4-16ug/ml), ceftazidime/avibactam (Avycaz, 8/4–16/4) ug/ml, tetracycline (TET, 4-16ug/ml), tegacycline (TGC, 0.5ug/ml), ciprofloxacin (CIP, 1-4ug/ml), nalidixic acid (NAL, 16-32ug/ml), azithromycin (AZM, 2ug/ml), amikacin (AMK, 16-64ug/ml), streptomycin (STR, 8-32ug/ml), ampicillin (AMP, 8-32ug/ml), ampicillin/sulbactam (SAM, 8/4–32/16ug/ml). The data was presented in three categories: susceptible (S), intermediate (I), and resistant (R).

### 2.3 DNA extraction

DNA were extracted with Bacterial Genomic DNA Extraction Kit (TIANGEN) according to the manufacturer’s instruction. The quality and concentration were determined using an Eppendorf BioSpectrometer® Basic. DNA was then stored at −20°C.

### 2.4 Genomic sequencing

Ninety-five isolates, representing over 40.00% of the total, were selected for their geographic diversity in Dapeng district. This group includes all 27 isolates from clinical patients, 47 isolates from seafood spanning fish, crustaceans, shellfish, and cephalopods, which are largely popular with the public, and 21 isolates from seawater samples across all collection sites along the Dapeng coastline (Supplementary Table 1). Extracted nucleic acids were quantified by QubitTMdsDNA HS Assay Kit (Thermo Fisher Scientific) and Qubit fluorometer (Thermo Fisher Scientific). Paired-end libraries were constructed with Illumina DNA Prep kit and the libraries were sequenced using 2x300bp paired end MiSeq Reagent Kit v3 (Illumina) on the Miseq sequencer (Illunina) with coverage >25×. Raw reads were trimmed and then *de novo* assembled using CLC Genomics Workbench version 23 (CLC Bio, Aarhus, Denmark).

### 2.5 Genetic analysis of antimicrobial resistance genes

ARGs were analyzed using Resfinder 4.1 with minimum sequence alignment coverage of 0.6 and sequence identity threshold of 0.8 (Florensa et al., 2022).

### 2.6 Genetic analysis of virulence

Hemolysin-producing genes *tdh, trh* and *tlh* were cloned and detected by PCR using *Vibrio parahaemolyticus* Triple Nucleic Acid-Detection Kit (MABSKY). Positive DNA controls for *trh*, *tlh* genes (CGMCC1.1997) and *tdh* gene (CGMCC1.1615) were included. The genes were identified by screening DNA sequences against core data set in Virulence Factor Database (VFDB; Chen et al., 2005). Minimum sequence alignment coverage and threshold for sequence identity are both 0.9.

### 2.7 SNP calling and phylogenetic tree construction

The SNP alignment was generated by aligning the reads to the core genome of *V. parahaemolyticus* useing strain RIMD2210633 as reference (Gonzalez-Escalona et al., 2017). A neighbor joining tree was constructed based on core genome SNPs using the Compare Variants Across Samples workflow in CLC Genomi 5cs Workbench version 23. The variant calling parameters were set to 10x minimum coverage, a minimum count of 10 and a 70% minimum frequency. For SNP tree creation, the parameters included 10x minimum coverage, 10% minimum frequency, a 0 prune distance, and the inclusion of multinucleotide variants (MNVs).

### 2.8 Statistical analysis

Statistical analysis was performed using Microsoft Office Excel 2010 and data are expressed as numbers or percentages of isolates.

## 3 Results

### 3.1 Distribution of *Vibrio parahaemolyticus* isolates in Dapeng

Of the 220 isolates, 27 originated from clinical patients in which 15 isolates were gathered from 3 foodborne outbreak cases in 2020 and 2023 while the others were contributed by hospital. Additionally, 134 isolates were obtained from seawater samples and another 59 isolates were collected from seafood. Specifically, 19 isolates were obtained from fish indicating 40.43% *V. parahaemolyticus* positivity. 14 isolates were sourced from 43 crustaceans samples (32.56%), including shrimps and crabs. 22 isolates were sourced from 51 shellfish samples (43.14%), comprising oysters, scallops, snails and abalones. The remaining 4 isolates were from 5 cephalopods samples (80.00%), including squids and octopus. Generally, 40.41% seafood were observed carrying *V. parahaemolyticus* (Table 1).

### 3.2 Antimicrobial susceptibilities

Among the 220 *V. parahemolyticus* isolates, 27 were retrieved from clinical patients, 59 were derived from seafood, and 134 were sourced from seawater. In order to uncover the antimicrobial resistance (AMR) profiles of these isolates, we performed a series of drug susceptibility tests (Table 2). Drug susceptibility tests demonstrated that among the 134 isolates derived from seawater, 73.88% isolates and 67.91% isolates exhibited high resistance to ampicillin and colistin, respectively. 32.09% of the isolates together with 57.46% isolates showed intermediate resistance to colistin and streptomycin. Low levels of resistance to cefotaxime and tetracycline were detected with the positivity of 1.49 and 4.48%. Furthermore, all isolates displayed sensitivity to additional 12 antibiotics, which were amikacin, azithromycin, chloramphenicol, ceftazidime, ciprofloxacin, ceftazidime avibactam, ertapenem, meropenem, nalidixic acid, ampicillin/sulbactam, compound sulfamethoxazole, and tigecycline.

**Table 2:** Antibiotic susceptibility [SIR*-No. of isolates (%)] of *V. parahaemolyticus* isolates.

Antibiotics	Abbreviation	Range (MIC, ug/mL)			Susceptibility no (%)											
					All samples			Patients			Seafood			Seawater		
		R	I	S	R	I	S	R	I	S	R	I	S	R	I	S
Chlorphenicol	CHL	≥32	16	≤8	0	0	220 (100.00%)	0	0	27 (100.00%)	0	0	59 (100.00%)	0	0	134 (100.00%)
Trimethoprim/Sulfamethoxazole	SXT	≥4/76	–	≤2/38	0	0	220 (100.00%)	0	0	27 (100.00%)	0	0	59 (100.00%)	0	0	134 (100.00%)
Colistin	COL	≥4	≤2	–	158 (71.82%)	62 (28.18%)	0	20 (74.07%)	7 (25.93%)	0	47 (79.66%)	12 (20.34%)	0	91 (67.91%)	43 (32.09%)	0
Ertapenem	ETP	≥2	1	≤0.5	0	0	220 (100.00%)	0	0	27 (100.00%)	0	0	59 (100.00%)	0	0	134 (100.00%)
Meropenem	MEM	≥4	2	≤1	0	0	220 (100.00%)	0	0	27 (100.00%)	0	0	59 (100.00%)	0	0	134 (100.00%)
Cefotaxime	CTX	≥4	2	≤1	4 (1.82%)	0	216 (98.18%)	0	0	27 (100.00%)	2 (3.39%)	0	57 (96.61%)	2 (1.49%)	0	132 (98.51%)
Ceftazidime	CAZ	≥16	8	≤4	0	0	220 (100.00%)	0	0	27 (100.00%)	0	0	59 (100.00%)	0	0	134 (100.00%)
Ceftazidime/avibactam	AVYCAZ	≥16/4	–	≤8/4	0	0	220 (100.00%)	0	0	27 (100.00%)	0	0	59 (100.00%)	0	0	134 (100.00%)
Tetracycline	TET	≥16	8	≤4	8 (3.64%)	0	212 (96.36%)	0	0	27 (100.00%)	2 (3.39%)	0	57 (96.61%)	6 (4.48%)	0	128 (95.52%)
Tegacycline	TGC	≥0.5	–	≤0.5	0	0	220 (100.00%)	0	0	27 (100.00%)	0	0	59 (100.00%)	0	0	134 (100.00%)
Ciprofloxacin	CIP	≥4	2	≤1	0	0	220 (100.00%)	0	0	27 (100.00%)	0	0	59 (100.00%)	0	0	134 (100.00%)
Nalidixic acid	NAL	≥32	–	≤16	0	0	220 (100.00%)	0	0	27 (100.00%)	0	0	59 (100.00%)	0	0	134 (100.00%)
Azithromycin	AZM	–	–	≤2	0	0	220 (100.00%)	0	0	27 (100.00%)	0	0	59 (100.00%)	0	0	134 (100.00%)
Amikacin	AMK	≥64	32	≤16	0	0	220 (100.00%)	0	0	27 (100.00%)	0	0	59 (100.00%)	0	0	134 (100.00%)
Streptomycin	STR	≥32	16	≤8	0	126 (57.27%)	94 (42.73%)	1 (3.70%)	24 (88.89%)	2 (7.41%)	0	25 (42.37%)	34 (57.63%)	0	77 (57.46%)	57 (42.54%)
Ampicillin	AMP	≥32	16	≤8	171 (77.73%)	1 (0.45%)	48 (21.82%)	27 (100.00%)	0	0	45 (76.27%)	1 (1.70%)	13 (22.03%)	99 (73.88%)	0	35 (26.12%)
Ampicillin/sulbactam	SAM	≥32/16	8/16	≤8/4	0	0	220 (100.00%)	0	0	27 (100.00%)	0	0	59 (100.00%)	0	0	134 (100.00%)

SIR*, susceptible, intermediate, resistant.

As for the bacteria collected from seafood, a fraction of which demonstrated resistance to cefotaxime (3.39%, *n* = 2) and tetracycline (3.39%, *n* = 2), while around 40% isolates showed resistance to streptomycin. Importantly, nearly 80% of isolates showed resistance to ampicillin and colistin, which was consistent with those in clinical samples. Meanwhile, 88.89% isolates exhibited strong resistance toward streptomycin.

Overall, antimicrobial resistance profiling revealed a consistent trend among isolates from various sources, with the majority exhibiting resistance to ampicillin and colistin, and intermediate level of resistance to streptomycin.

### 3.3 Analysis of antimicrobial resistance genes

Resistance toward *blaCARB, tet(34), tet(35), qnrVC6*, *sul2*, *aph(6)-Id*, *floR* and *cat* was identified (Table 3). *blaCARB* encodes *β*-lactamase enzyme and confers resistance to amoxicillin, ampicillin, and piperacillin (Shallal et al., 2019). Meanwhile, *tet(34)* and *tet(35)* are known to mediate tetracycline resistance (Miranda et al., 2003). These three genes were determined positive in all clinical and environmental isolates. No additional ARGs were identified within clinical samples. Furthermore, the positive rates of *qnrVC6*, *sul2*, *aph(6)-Id*, *floR* and *cat* were ranged from 2% ~12% in environmental isolates collected in Dapeng. The positivity exhibited lower value when it came to the isolates aside of Dapeng. Notably, a single isolate was positive for the *blaCTX-M-14*, which encodes extended-spectrum *β*-lactamase.

**Table 3:** Antimicrobial resistance gene (ARG) profiles of 218 *V. parahaemolyticus* genomes detected with ResFinder.

Antimicrobial classes	Genes	Other areas in Shenzhen		Dapeng			
		123 genomes (Environment)		68 genomes (Environment)		27 genomes (Patients)	
		Number of positive genomes	Percentage (%)	Number of positive genomes	Percentage (%)	Number of positive genomes	Percentage (%)
β-lactam	*blaCARB*	123	100.00	68	100.00	27	100.00
	*blaCTX-M-14*	0	0	1	1.00	0	0
Tetracycline	*tet(35)*	123	100.00	68	100.00	27	100.00
	*tet(34)*	123	100.00	68	100.00	27	100.00
	*tet(59)*	2	1.63	0	0	0	0
	*tet(E)*	0	0	1	1.00	0	0
	*tet(S/M)*	0	0	2	3	0	0
Quinolone	*qnrVC6*	4	3.25	3	4	0	0
Trimethoprim	*dfrA6*	4	3.25	0	0	0	0
Sulphonamide	*sul2*	3	2.44	7	10	0	0
Fosfomycin	*fos*	2	1.63	0	0	0	0
Aminoglycoside	*aph(6)-Id*	1	0.81	4	6	0	0
Phenicol	*floR*	1	0.81	5	7	0	0
	*cat*	0	0	2	3	0	0

### 3.4 Identification of virulence factors

The results revealed that all 220 isolates from the Dapeng district tested positive for the *tlh* gene. 193 environmental isolates were negative for the *tdh* or *trh* gene. The *tdh* gene was consistently detected in the 27 clinical isolates, with one isolate exhibiting both *tdh* and *trh* genes (Table 4).

**Table 4:** Virulence factor profiles of 218 *V. parahaemolyticus* genomes detected with VFDB.

Virulence factors			Tdh	Trh	MAM7	T3SS1	T3SS1 secreted effector	T3SS2	T3SS2 secreted effector	VpadF
Dapeng	68 genomes (Environment)	Number of positive genomes	0	0	61	68	68	1	0	59
		Percentage (%)	0	0	89.71	100.00	100.00	1.47	0.00	86.76
	27 genomes (Patients)	Number of positive genomes	27	1	27	27	27	26	26	26
		Percentage (%)	100.00	3.70	100.00	100.00	100.00	96.30	96.30	96.30
Other areas in Shenzhen	123 genomes (Environment)	Number of positive genomes	2	0	123	123	122	2	2	107
		Percentage (%)	1.63	0.00	100.00	100.00	99.19	1.63	1.63	86.99

Six virulence factors were detected and then analyzed. MAM7, T3SS1, T3SS1 secret effectors were detected positive in all clinical samples, so were the sample derived from environment while the MAM7 positive rate decreased to 89.71%. Meanwhile, 96.30% isolates were positive for T3SS2, T3SS2 secret effectors and VpadF. Notably, only one isolate contained the T3SS2 factor.

Notably, none isolates derived from seafood in other districts were found harboring *trh* genomes. Besides, two *tdh*-positive isolates were observed to carry both T3SS2 and T3SS2 secret effectors. Additionally, MAM7 together with T3SS1 presented in all samples while T3SS2 and its secret effectors were merely found (Figure 1; Table 4; Supplementary Table 1).

### 3.5 Phylogenomic relationship of *Vibrio parahaemolyticus*

Based on WGS, phylogenomic analysis disclosed that isolates from the three foodborne outbreak cases could be generally clustered into three separate sub-clades according to average genetic distance (Figure 1). In detail, the SNPs differences of genomes and isolates under 10 and 2000, respectively, were defined as genetically sub-clade (Wu et al., 2019). As for case 1 and 3, no SNP difference were observed among their isolates, respectively. Notably, in case 2, isolate JS200829003 harbored both *tdh* and *trh* genes, was divided into a distinct clade (Supplementary Table 2).

**Figure 2:**
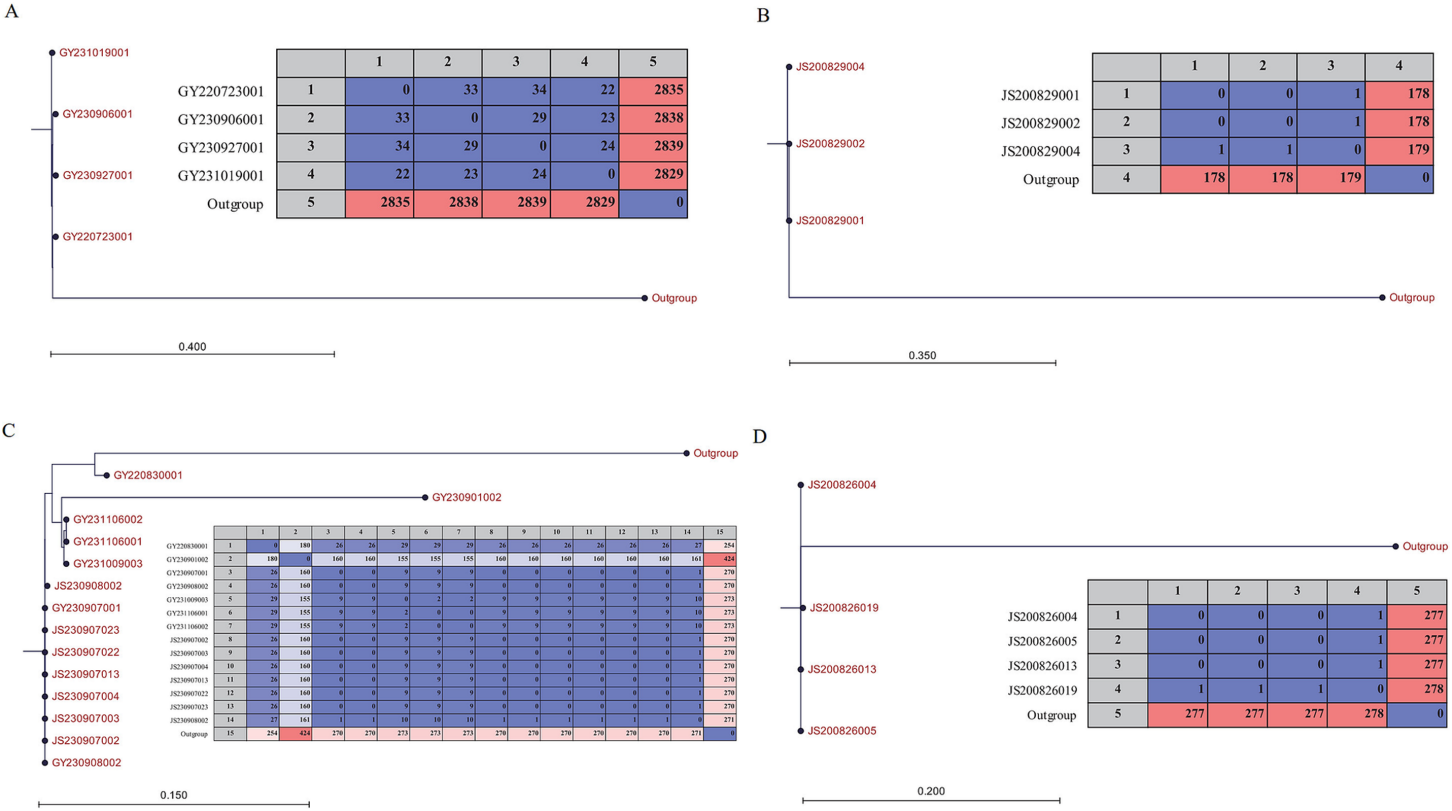
Matrix and Neighbor-Joining phylogeny based on SNPs of the *V. parahaemolyticus* isolates which could be from 4 different reservoirs, respectively. (A) Four isolates collected from IDDS. (B) and (D) Isolates collected from two different food-borne outbreak cases. (C) Isolates collected from IDDS and food-borne outbreak case. The scale bar indicates branch lengths within the tree.

The 12 isolates obtained hospital were divided into three sub-clade (Figure 1). The size of the core genome was inversely proportional to the number of isolates, which in turn influenced the extent of the SNP (Yang et al., 2019). SNP-trees were reconstructed within each sub-clade since the reassessment of SNPs was expected to achieve higher resolution. The results indicated that the SNP differences among isolates from the same foodborne outbreak case were ≤ 1, which was consistent with the findings presented in Figure 1. Compared to the isolates in case 3, no SNP difference was observed in two isolates from IDDS program while the other five isolates valued up to 29. Furthermore, among the four isolates clustered within a sub-clade, the SNP differences were ≤ 34 (Figure 2).

Excluding JS200829003, all clinical isolates were found to be clustered within a same clade, together with another two environmental isolates from other districts. Meanwhile, these two environmental isolates also exhibited close genetic proximity to three isolates from case 2 as well as part of isolates obtained from hospital. Other isolates from environment were almost scattered across different clades (Figure 1), suggesting a high degree of genetic diversity. The distinct sampling time suggested a persistent environmental reservoir of pathogenic *V. parahaemolyticus*.

## 4 Discussion

*Vibrio parahaemolyticus* contamination was determined to exist in seafood and seawater widely in Shenzhen, which was consistent with the report of WHO (WHO, 2021). The incidence rate of *V. parahaemolyticus* in seafood was 40.41%, which is lower compared to previous reports from the eastern coastline of Jiangsu, China, where it was 59% (Zhao et al., 2011). This phenomenon was possibly contributed by the decrease in domestic wastewater which was led by strict environment protection measures, as the nontoxic and nutritious domestic sewage could promote microbial biodiversity in aquatic ecosystems (Abioye et al., 2021; Li et al., 2017). However, the positivity of *V. parahaemolyticus* in Dapeng seawaterthe demonstrated a significant higher value compared to that in freshwater (Chao et al., 2009). Thus, the influence of temperature and salinity on *Vibrio* density and frequency should also be taken into consideration (Jordaan et al., 2019).

Previous studies have reported that *V. parahaemolyticus* exhibits multiple antibiotic resistance to multiple antibiotic agents (Pan et al., 2013; Tan et al., 2020; Zaafrane et al., 2022; Zhang et al., 2024). Though it was reported that the *V. parahaemolyticus* resistance to ampicillin has been decreasing in Shenzhen (Zhang et al., 2023), high prevalence of AMR was observed with 77.73% for ampicillin and 71.82% for colistin (Table 2), suggested that ampicillin and colistin should not be used empirically to treat *V. parahaemolyticus* infection. Interestingly, the phenotype of resistance to ampicillin was significantly correlated with the genotype of the isolates, which were positive for *blaCARB*. However, no corresponding genes for resistance to colistin and streptomycin were observed among the 218 genomes (Tables 2, 3), which could be related to the inability of short-read sequencing in identifying mobile genetic elements (Dutta et al., 2021). Approximately all isolates from Dapeng carried both *tet(35)* and *tet(34)* genes and were susceptible to tetracycline. It is plausible that these genes were silenced or expressed at a level too low to be detected, and could be highly expressed under suitable conditions (Dutta et al., 2021). Thus, the diversity of ARGs and virulence genes in the environment is a vital factor for the risk assessment of *V. parahaemolyticus.* The *dfrA6* and *fos* genes were only detected in the environment from other districts, but with very low incidences. Our study showed that the first-line drugs such as tetracycline, cefotaxime and ceftazidime can still be used to treat *V. parahaemolyticus* infection.

*tdh and trh genes* were once considered as the pathogenic marker of *V. parahaemolyticus* (Raghunath, 2014), however only part of clinical isolates were *tdh* gene positive and one clinical isolate carried both *tdh* and *trh,* indicating that pathogenicity could be caused by other virulence factors (Jones et al., 2012; Li et al., 2014; Pazhani et al., 2014). Thus, MAM7, T3SS1, T3SS1 secret effects, T3SS2, T3SS2 secret effects, and Vpad F were then identified in the isolates which were *tdh* gene positive. It was reported that T3SS2 was only present in the *tdh*-positive isolates (Jones et al., 2012) and genes expressing T3SS2 were adjacent to *tdh* and *trh* genes (Yang et al., 2019). However, aside of T3SS2, the isolate carrying both the *tdh* and *trh* lacked T3SS2 secret effectors and VpadF additionally. Interestingly, it’s opposite one, a *tdh^−^trh^−^* isolate possessed T3SS2 and its secret effectors, suggesting horizontal transfer had occurred (Meador et al., 2007). Besides, almost all environmentally resourced isolates possessed MAM7, T3SS1, T3SS1 secret effectors and VpadF, demonstrating that the virulence genes were widely distributed, which could be activated under proper circumstances and pose threat to public health.

To characterize the phylogenomic relationship of *V. parahaemolyticus* isolates from various resources and locations, core-genomes of isolates were analyzed (Gonzalez-Escalona et al., 2017). Isolates of three foodborne outbreak cases were clustered into different sub-clades within which the SNP difference was less than 1 (Figures 1, 2). Thus, this result further confirmed that the core genome is a powerful tool for outbreak investigation, allowing for the unambiguous comparison of isolates from different sources (Gonzalez-Escalona et al., 2017). Furthermore, JS200829003 collected from case 2 had a great genetic distance from others and showed different profile of virulence factors as described above, indicating it could be originated from other sources. Meanwhile, section of isolates from sporadic cases were clustered together with isolates from case 3 in a sub-clade (Figure 1). Further analysis showed that the SNP differences between these 14 isolates slightly increased (Figure 2C), suggesting that they were from an another reservoir.

The dates of collection of the two isolates from IDDS in the sub-clade were consistent with the outbreak date of case 3, with no SNP difference, indicating that this two patients could be originated from the foodborne outbreak in case 3. Perhaps these two patients were overlooked during the case search because they were treated in another different hospital. Other five isolates from sporadic cases were collected from 2022 to 2023 years, indicating that reservoir 3 already existed in 2022 (Figures 1, 2C). In August 2020, two foodborne outbreak cases caused by *V. parahaemolyticus* occurred. The phylogenetic tree showed that the sources of these two cases were from different reservoirs. However, no environmental isolates from Dapeng coastline was clustered with clinical isolates together within a same clade. Certainly, one possible reason could be the limited number of bacterial genomes sequenced in this study, implying that pathogenic isolates may come from other districts. According to the clonal lineage analysis based on GenBank (Yang et al., 2022), two isolates from seafood and the clinical isolates from Dapeng coastline were clustered in the same clade, with a difference of 116 ~ 397 SNPs (Supplementary Table 2). Ocean currents and aquatic animals contribute to the dissemination of *V. parahaemolyticus*, and genetic exchange frequently occurs when isolates enter a new environment, greatly accelerating the mixing of *V. parahaemolyticus* populations (Fu et al., 2021). So it is reasonable to speculate that the isolates clustered in clade 2 could be from the same clone, and the pathogen *V. parahaemolyticus* were transported from other districts of Shenzhen to Dapeng coastline.

Almost all the isolates collected from environment were distributed in different clades, with no obvious clustering in the NJ tree. The abundant genomic polymorphism was helpful for *Vibrio parahaemolyticus* to adapt to the changing external environment (Janecko et al., 2021). While almost all clinical isolates clustered in clade 2, inferring that a stable genomic with less recombination and variation is vital for clinical isolates to maintain pathogenicity (He et al., 2021). Some environmental isolates were clustered in the same clade or sub-clade, which suggested they could be from the same source. This possibility is considered due to factors like ocean currents, dispersal by aquatic animals, cross-contamination during seafood processing, and the long-term survival of these isolates in the environment (Fu et al., 2021). Overall, the phylogenomic analysis revealed that *V. parahaemolyticus* isolates in environment of Shenzhen were genetically diverse.

There are several limitations in our study. Firstly, the period of surveillance is relatively short, thus the results may not reflect the long-term evolution of *V. parahaemolyticus* in Dapeng coastline. In the future, constant surveillance should be carried to monitor the evolution trend of *V. parahaemolyticus* in the Dapeng coastline. Additionally, the distribution of *V. parahaemolyticus* isolates included in this study did not cover all districts of Shenzhen over a continuous period in recent years. Therefore, the dynamics of *V. parahaemolyticus* populations in Shenzhen city need further research. Associated studies may provide clues for future work.

In conclusion, this study revealed the characteristic of *V. parahaemolyticus* isolates in Dapeng coastline. The widely distribution of *V. parahaemolyticus* isolates poses the risk to the health to the population living in coastline and even hinterland. Moreover, AMR result of *V. parahaemolyticus* provides instruction for empirically treatment in patients. Considering the inducible gene expression, horizontal gene transfer and the diverse ARGs and virulence genes harbored in *V. parahaemolyticus*, it should be paid more attention to monitoring and predicting the changes and development tendency of the pathogen. Phylogenomic analysis indicates that several reservoirs hidden in environment, and hints the pathogen isolates may be disseminated though various pathways involving ocean currents, aquatic animals and cross-contamination during seafood processing, providing a significant insight into the prediction and interruption of *V. parahaemolyticus* transmission routes.

## Data Availability

The datasets presented in this study can be found in online repositories. The names of the repository/repositories and accession number(s) can be found in the article/Supplementary material.

## References

[ref1] Abioye O. E. Osunla A. C. Okoh A. I. (2021). Molecular detection and distribution of six medically important Vibrio spp. in selected freshwater and brackish water resources in eastern Cape Province, South Africa. Front. Microbiol. 12:617703. doi: 10.3389/fmicb.2021.617703, PMID: 34149632 PMC8208477

[ref2] Ansari M. I. Grohmann E. Malik A. (2008). Conjugative plasmids in multi-resistant bacterial isolates from Indian soil. J. Appl. Microbiol. 104, 1774–1781. doi: 10.1111/j.1365-2672.2008.03736.x, PMID: 18284489

[ref3] Baker-Austin C. Oliver J. D. Alam M. Ali A. Waldor M. K. Qadri F.. (2018). Vibrio spp. infections. Nat. Rev. Dis. Primers 4:8. doi: 10.1038/s41572-018-0005-8, PMID: 30002421

[ref4] Baker-Austin C. Stockley L. Rangdale R. Martinez-Urtaza J. (2010). Environmental occurrence and clinical impact of Vibrio vulnificus and *Vibrio parahaemolyticus*: a European perspective. Environ. Microbiol. Rep. 2, 7–18. doi: 10.1111/j.1758-2229.2009.00096.x, PMID: 23765993

[ref5] Cabello F. C. (2006). Heavy use of prophylactic antibiotics in aquaculture: a growing problem for human and animal health and for the environment. Environ. Microbiol. 8, 1137–1144. doi: 10.1111/j.1462-2920.2006.01054.x, PMID: 16817922

[ref6] Chao G. Jiao X. Zhou X. Yang Z. Huang J. Zhou L.. (2009). Distribution, prevalence, molecular typing, and virulence of *Vibrio parahaemolyticus* isolated from different sources in coastal province Jiangsu, China. Food Control 20, 907–912. doi: 10.1016/j.foodcont.2009.01.004

[ref7] Chen L. Yang J. Yu J. Yao Z. Sun L. Shen Y.. (2005). VFDB: a reference database for bacterial virulence factors. Nucleic Acids Res. 33, D325–D328. doi: 10.1093/nar/gki008, PMID: 15608208 PMC539962

[ref8] CLSI, Clinical and Laboratory Standards Institute (2024). Performance standards for antimicrobial susceptibility testing: CLSI supplement M100. 33rd Edn. PA, USA: Clinical and Laboratory Standards Institute (CLSI), Wayne.

[ref9] Dutta D. Kaushik A. Kumar D. Bag S. (2021). Foodborne pathogenic vibrios: antimicrobial resistance. Front. Microbiol. 12:638331. doi: 10.3389/fmicb.2021.638331, PMID: 34276582 PMC8278402

[ref10] Elmahdi S. DaSilva L. V. Parveen S. (2016). Antibiotic resistance of Vibrio parahaemolyticus and *Vibrio vulnificus* in various countries: a review. Food Microbiol. 57, 128–134. doi: 10.1016/j.fm.2016.02.008, PMID: 27052711

[ref11] Florensa A. F. Kaas R. S. Clausen P. T. L. C. Aytan-Aktug D. Aarestrup F. M. (2022). ResFinder–an open online resource for identification of antimicrobial resistance genes in next-generation sequencing data and prediction of phenotypes from genotypes. Microbial Genomics 8:000748. doi: 10.1099/mgen.0.000748, PMID: 35072601 PMC8914360

[ref12] Fu S. Wang Q. Zhang Y. Yang Q. Hao J. Liu Y.. (2021). Dynamics and microevolution of *Vibrio parahaemolyticus* populations in shellfish farms. mSystems 6:6. doi: 10.1128/mSystems.01161-20, PMID: 33436516 PMC7901483

[ref13] Gonzalez-Escalona N. Jolley K. A. Reed E. Martinez-Urtaza J. (2017). Defining a Core genome multilocus sequence typing scheme for the global epidemiology of *Vibrio parahaemolyticus*. J. Clin. Microbiol. 55, 1682–1697. doi: 10.1128/jcm.00227-17, PMID: 28330888 PMC5442524

[ref14] He M. Lei T. Jiang F. Zhang J. Zeng H. Wang J.. (2021). Genetic diversity and population structure of *Vibrio parahaemolyticus* isolated from clinical and food sources. Front. Microbiol. 12:708795. doi: 10.3389/fmicb.2021.708795, PMID: 34385993 PMC8353399

[ref15] Janecko N. Bloomfield S. J. Palau R. Mather A. E. (2021). Whole genome sequencing reveals great diversity of Vibrio spp in prawns at retail. Microb Genom 7, 1–14. doi: 10.1099/mgen.0.000647, PMID: 34586050 PMC8715430

[ref16] Jin J. Zhou Y. Zhang Z. Wang H. Hou W. Wang H.. (2021). Characteristics of antimicrobial-resistant *Vibrio parahaemolyticus* strains and identification of related antimicrobial resistance gene mutations. Foodborne Pathog. Dis. 18, 873–879. doi: 10.1089/fpd.2020.2911, PMID: 34279997

[ref17] Jones J. L. Lüdeke C. H. M. Bowers J. C. Garrett N. Fischer M. Parsons M. B.. (2012). Biochemical, serological, and virulence characterization of clinical and oyster *Vibrio parahaemolyticus* isolates, vol. 50, 2343–2352. doi: 10.1128/JCM.00196-12 PMC3405591 22535979

[ref18] Jordaan K. Comeau A. M. Khasa D. P. Bezuidenhout C. C. (2019). An integrated insight into the response of bacterial communities to anthropogenic contaminants in a river: a case study of the Wonderfonteinspruit catchment area, South Africa. PLoS One 14:e0216758. doi: 10.1371/journal.pone.0216758, PMID: 31112559 PMC6528982

[ref19] Letchumanan V. Chan K. G. Lee L. H. (2014). *Vibrio parahaemolyticus*: a review on the pathogenesis, prevalence, and advance molecular identification techniques. Front. Microbiol. 5:705. doi: 10.3389/fmicb.2014.00705, PMID: 25566219 PMC4263241

[ref20] Li D. Jiang X. Wang J. Wang K. Zheng B. (2017). Effect of sewage and industrial effluents on bacterial and archaeal communities of creek sediments in the Taihu Basin. WaterSA 9, 1–19. doi: 10.3390/w9060373

[ref21] Li Y. Xie X. Shi X. Lin Y. Qiu Y. Mou J.. (2014). *Vibrio parahaemolyticus*, southern coastal region of China, 2007-2012. Emerg. Infect. Dis. 20, 685–688. doi: 10.3201/eid2004.130744, PMID: 24655369 PMC3966377

[ref22] Malik A. Aleem A. (2011). Incidence of metal and antibiotic resistance in Pseudomonas spp. from the river water, agricultural soil irrigated with wastewater and groundwater. Environ. Monit. Assess. 178, 293–308. doi: 10.1007/s10661-010-1690-2, PMID: 20853188

[ref23] Mazel D. Davies J. (1999). Antibiotic resistance in microbes. Cell. Mol. Life Sci. 56, 742–754. doi: 10.1007/s000180050021, PMID: 11212334 PMC11147152

[ref24] Meador C. E. Parsons M. M. Bopp C. A. Gerner-Smidt P. Painter J. A. Vora G. J. (2007). Virulence gene-and pandemic group-specific marker profiling of clinical *Vibrio parahaemolyticus* isolates. J. Clin. Microbiol. 45, 1133–1139. doi: 10.1128/JCM.00042-07, PMID: 17301274 PMC1865801

[ref25] Miranda C. D. Kehrenberg C. Ulep C. Schwarz S. Roberts M. C. (2003). Diversity of tetracycline resistance genes in bacteria from Chilean salmon farms. Antimicrob. Agents Chemother. 47, 883–888. doi: 10.1128/AAC.47.3.883-888.2003, PMID: 12604516 PMC149303

[ref26] Morris J. G. Jr. Tenney J. (1985). Antibiotic therapy for *Vibrio vulnificus* infection. JAMA 253, 1121–1122. doi: 10.1001/jama.1985.03350320041011 3968842

[ref27] Nishibuchi M. Kaper J. B. (1995). Thermostable direct hemolysin gene of *Vibrio parahaemolyticus*: a virulence gene acquired by a marine bacterium. Infect. Immun. 63, 2093–2099. doi: 10.1128/iai.63.6.2093-2099.1995, PMID: 7768586 PMC173271

[ref28] Nordstrom J. L. Vickery M. C. Blackstone G. M. Murray S. L. DePaola A. (2007). Development of a multiplex real-time PCR assay with an internal amplification control for the detection of total and pathogenic *Vibrio parahaemolyticus* bacteria in oysters. Appl. Environ. Microbiol. 73, 5840–5847. doi: 10.1128/aem.00460-07, PMID: 17644647 PMC2074920

[ref29] Pan J. Zhang Y. Jin D. Ding G. Luo Y. Zhang J.. (2013). Molecular characterization and antibiotic susceptibility of *Vibrio vulnificus* in retail shrimps in Hangzhou. People's Republic of China, J Food Prot 76, 2063–2068. doi: 10.4315/0362-028x.Jfp-13-161, PMID: 24290683

[ref30] Pazhani G. P. Bhowmik S. K. Ghosh S. Guin S. Dutta S. Rajendran K.. (2014). Trends in the epidemiology of pandemic and non-pandemic strains of *Vibrio parahaemolyticus* isolated from diarrheal patients in Kolkata. India, PLoS Negl Trop Dis 8:e2815. doi: 10.1371/journal.pntd.0002815, PMID: 24786538 PMC4006737

[ref31] Raghunath P. (2014). Roles of thermostable direct hemolysin (TDH) and TDH-related hemolysin (TRH) in *Vibrio parahaemolyticus*. Front. Microbiol. 5:805. doi: 10.3389/fmicb.2014.00805, PMID: 25657643 PMC4302984

[ref32] Ralph A. Currie B. J. (2007). Vibrio vulnificus and *V. parahaemolyticus* necrotising fasciitis in fishermen visiting an estuarine tropical northern Australian location. J. Inf. Secur. 54, e111–e114. doi: 10.1016/j.jinf.2006.06.015, PMID: 16890991

[ref33] Shallal Z. S. AL-Suraifi A. S. K. Hadil A. L.-H. (2019). Detection of extended Spectrum β-lactamase (ESBL) amongGram-negative bacteria isolates from workers in a restaurant in Wasit province, Iraq. J. Pharmaceutical Sci. Res. 11, 1602–1609.

[ref34] Su Y.-C. Liu C. (2007). *Vibrio parahaemolyticus*: a concern of seafood safety. Food Microbiol. 24, 549–558. doi: 10.1016/j.fm.2007.01.005 17418305

[ref35] Tan C. W. Rukayadi Y. Hasan H. Thung T. Y. Lee E. Rollon W. D.. (2020). Prevalence and antibiotic resistance patterns of *Vibrio parahaemolyticus* isolated from different types of seafood in Selangor, Malaysia. Saudi J Biol Sci 27, 1602–1608. doi: 10.1016/j.sjbs.2020.01.002, PMID: 32489301 PMC7253911

[ref36] World Health Organization & Food and Agriculture Organization of the United Nations. (2021). Advances in science and risk assessment tools for Vibrio parahaemolyticus and V. vulnificus associated with seafood: meeting report. World Health Organization. https://www.who.int/publications/i/item/9789240024878

[ref37] Wu K. Zheng Y. Qingping W. Chen H. Songzhe F. Kan B.. (2019). *Vibrio parahaemolyticus* cqsA controls production of quorum sensing signal molecule 3-hydroxyundecan-4-one and regulates colony morphology. Misaengmul Hakhoe chi 57, 1105–1114. doi: 10.1007/s12275-019-9379-x 31686391

[ref38] Yang C. Li Y. Jiang M. Wang L. Jiang Y. Hu L.. (2022). Outbreak dynamics of foodborne pathogen *Vibrio parahaemolyticus* over a seventeen year period implies hidden reservoirs. Nat. Microbiol. 7, 1221–1229. doi: 10.1038/s41564-022-01182-0, PMID: 35918422

[ref39] Yang C. Zhang X. Fan H. Li Y. Hu Q. Yang R.. (2019). Genetic diversity, virulence factors and farm-to-table spread pattern of *Vibrio parahaemolyticus* food-associated isolates. Food Microbiol. 84:103270. doi: 10.1016/j.fm.2019.103270, PMID: 31421783

[ref40] Zaafrane S. Maatouk K. Alibi S. Ben Mansour H. (2022). Occurrence and antibiotic resistance of *Vibrio parahaemolyticus* isolated from the Tunisian coastal seawater. J. Water Health 20, 369–384. doi: 10.2166/wh.2022.243, PMID: 36366993

[ref41] Zhang J. Haochuan C. Runli C. Jiahao W. Lingling L. Song L. (2023). Analysis of virulence genes and drug resistance of *Vibrio parahaemolyticus* in aquatic products and aquaculture water in Shenzhen City from 2015-2020. Occup and Health 39, 3011–3014. doi: 10.13329/j.cnki.zyyjk.2023.0535

[ref42] Zhang F. Zhang J. Lin G. Chen X. Huang H. Xu C.. (2024). Antibiotic resistance and genetic profiles of *Vibrio parahaemolyticus* isolated from farmed Pacific white shrimp (*Litopenaeus vannamei*) in Ningde regions. Microorganisms 12, 1–11. doi: 10.3390/microorganisms12010152, PMID: 38257979 PMC10821069

[ref43] Zhao F. Zhou D.-q. Cao H.-h. Ma L.-p. Jiang Y.-h. (2011). Distribution, serological and molecular characterization of *Vibrio parahaemolyticus* from shellfish in the eastern coast of China. Food Control 22, 1095–1100. doi: 10.1016/j.foodcont.2010.12.017

